# Enteropathic Spondyloarthritis: From Diagnosis to Treatment

**DOI:** 10.1155/2013/631408

**Published:** 2013-04-15

**Authors:** Rosario Peluso, Matteo Nicola Dario Di Minno, Salvatore Iervolino, Francesco Manguso, Giuseppina Tramontano, Pasquale Ambrosino, Carmela Esposito, Antonella Scalera, Fabiana Castiglione, Raffaele Scarpa

**Affiliations:** ^1^Rheumatology Research Unit, University Federico II, 80131 Naples, Italy; ^2^Department of Clinical and Experimental Medicine, University Federico II, 80131 Naples, Italy; ^3^Regional Reference Center for Coagulation Disorders, University Federico II, 80131 Naples, Italy; ^4^Complex Operating Unit of Gastroenterology, AORN “A.Cardarelli,” Via Sergio Pansini 5, 80131 Naples, Italy; ^5^Gastroenterology Research Unit, University Federico II, 80131 Naples, Italy

## Abstract

Enteropathic arthritis (EA) is a spondyloarthritis (SpA) which occurs in patients with inflammatory bowel diseases (IBDs) and other gastrointestinal diseases. Diagnosis is generally established on the medical history and physical examination. It was, generally, made according to the European Spondyloarthropathy Study Group (ESSG) criteria. Rheumatic manifestations are the most frequent extraintestinal findings of IBD with a prevalence between 17% and 39%, and IBD is associated, less frequently, with other rheumatic disease such as rheumatoid arthritis, Sjogren syndrome, Takayasu arteritis, and fibromyalgia. Although the pathogenesis of EA has not been plainly clarified, the most popular theory supposes that joint inflammation occurs in genetically predisposed subjects with bacterial gut infections, provided an important evidence for a possible relationship between inflammation of the gut mucosa and arthritis. The management of patients with EA requires an active cooperation between the gastroenterologist and rheumatologist.

## 1. Introduction

Enteropathic arthritis or enteroarthritis (EA) is a spondyloarthritis (SpA) which occurs in patients with inflammatory bowel diseases (IBDs) and other gastrointestinal diseases, such as Whipple's disease (WD), celiac disease (CD), and intestinal bypass surgery [1, 2]. 

A relationship between bowel and joints was reported for the first time by Smith in 1922, who described in patients with rheumatoid arthritis (RA) underwent surgery for colectomy an improvement of articular symptoms [3]. Later, Bargen et al. [4], in 1929, and Hench [5], in 1935, described a peripheral arthritis involvement in patients with IBD and also reported the arthritis tendency to flare with exacerbation of the colitis and to recede with the remission of bowel symptoms. At the end of the 1950s, some authors described the occurrence of sacroiliitis in patients with UC [6] and CD [7–9]. Finally, in 1964, the American Rheumatism Association classified arthritis associated with IBD as independent clinical form [10], and, later, Wright and Moll included enteroarthritis definitively among SpA group [11]. In the group of enteropathic spondyloarthritis, more lately, the rare Whipple's disease [12, 13] and postenteritis reactive forms [14, 15] were also included. 

The aim of this review is to describe clinical and pathophysiological data about EA. However, because of the significant lack of studies on this specific issue, most of results are derived from studies on IBD or other types of spondyloarthritis. 

## 2. Classification Criteria

Diagnosis is generally established on the medical history and physical examination, because at present no “gold standard” criteria is available for the diagnosis of EA. Thus, being the SpA a group of distinct diseases with similar clinical features and a common genetic predisposition [16], the diagnosis of EA was, generally, made according to the European Spondyloarthropathy Study Group (ESSG) criteria [17]. In fact, IBD is a criterion of SpA; thus, patients with IBD presenting with inflammatory back pain and/or synovitis (predominantly of the lower limbs) are diagnosed as having spondyloarthropathy. These criteria, although they are not defined for diagnostic purposes, may be a useful guide for the clinician in the identification of patients with EA. Moreover, the ESSG criteria, designed to be applicable without radiological examination and laboratory testing, have good sensitivity (86%) and specificity (87%), at least in established disease.

## 3. Epidemiology

The results from epidemiologic studies on AE have been influenced by several factors, including the lack of validated sets of diagnostic criteria, the frequency of IBD in different geographic areas, the age cut-off and case definition, and different study designs. The incidence and prevalence of IBD in Western Countries is estimated to be 6-15/100,000 and 50-200/100,000, respectively, for CD, and 8-14/100,000 and 120-200/100,000, respectively, for UC [18]. 

Rheumatic manifestations are the most frequent extraintestinal manifestation in IBD patients with a prevalence ranging between 17% and 39% [19, 20] Interestingly, articular alterations can be diagnosed before, simultaneously, or after the diagnosis of IBD. The joint involvement observed in IBD is usually classified in two subsets: axial (including sacroiliitis with or without spondylitis) and peripheral. The axial involvement is found to be present in 2%–16% of IBD patients, with a higher prevalence in CD patients than in UC ones. Moreover, the prevalence of sacroiliitis (asymptomatic and symptomatic) is between 12% and 20% and association with HLA-B27 ranged from 3.9% to 18.9% [19]. Recently, some studies showed that the prevalence of axial joint involvement was higher than those reported previously, as already described by Scarpa et al. in 1992 [21]. In fact, in these studies, based on the ESSG criteria for SpA [17], the authors detected a frequency ranging between 10%–25% for spondylitis and 30%–36% for sacroiliitis [20, 22, 23].

The peripheral involvement is a common complication in both CD and UC and its prevalence has been reported in a wide range (0.4%–34.6%) of patients with IBD. It is reported to be more frequent in CD than UC (20% and 10%, resp.) [24] and it predominantly affects the joints of the lower limbs [25]. Women show more frequently a peripheral joint involvement, whereas men tend to have an axial involvement [26, 27]. 

Interestingly, potential risk factors for arthritis in IBD patients are active bowel disease, family history of IBD, appendectomy, cigarette smoking, and the presence of others extraintestinal manifestations, such as erythema nodosum or pyoderma gangrenosum [28–31].

## 4. Pathogenesis

Although the pathogenesis of EA has not been plainly clarified, the observation that joint inflammation occurs in genetically predisposed subjects with bacterial gut infections provided an important evidence for a possible relationship between inflammation of the gut mucosa and arthritis. At the end of the 80s, some authors described that more than two-third of patients with SpA showed microscopic inflammatory changes of gut mucosa without clinical signs of gastrointestinal disease [32–35]. In 2000, Scarpa et al. described microscopic lesions of gut mucosa in PsA patients without bowel symptoms, also when mucosa appeared macroscopically normal, suggesting a pathogenetic link between joints, gut and skin in PsA patients [36].

Current theories provide, in genetically predisposed subjects, an aberrant migration of intestinal lymphocytes or macrophages from inflamed gut mucosa to joints, in which an important role was played by gut bacteria. In fact, a dysfunctional interaction between the mucosal immune system and gut bacteria could result in an abnormal state of immunological tolerance toward flora by alterations in mucosal effector cells or by affecting regulatory cells [37]. A significant evidence for the pathogenic role of gut bacteria in the pathogenesis of SpA is derived from animals models. 

In details, in the HLA-B27/human *β*2-microglobulin transgenic rat model, a high copy number of the transgene is necessary to induce an SpA like disease, characterized the development of arthritis (sacroiliitis, spondylitis, and peripheral arthritis) and extra-articular manifestations (psoriasiform skin, nail lesions, and enterocolitis). In addition, these rats do not develop joint, skin, and gut inflammation when kept in germ-free conditions, reflecting the interplay between predisposing genes and gut bacteria [38]. 

In this complex scenario, genetic factors play a predisposing role while environmental factors, such as infectious agents, may play a causative role.

Among the genetic factors, HLA-B27 has the strongest genetic association with SpA and in particular with ankylosing spondylitis (AS) [39]. This association is reported in more than 90% of cases [40]. Also spondylitis in IBD is associated with the presence of HLA-B27, however, in lower frequencies than in AS (30%–80%) [24, 41]. Pure asymptomatic sacroiliitis in CD is not strongly associated with HLA-B27 and a very recent study indicates a prevalence of 7% [42]. In 2000, Orchard et al. described an association with HLA-DR0103, B35, and B27 in type 1 peripheral arthritis and neither B27 nor DR4 associations were observed in type 2 arthritis [43]. 

In order to describe the role of HLA-B27 in the pathogenesis of EA, different theories have been proposed. 

One theory suggests that HLA-B27-expressing macrophages expose specific bacterial antigens that may activate CD4+ T-cells with their migration from gut to joint with development of arthritis [44–46]. Another theory proposes that homology between HLA-B27 sequences and bacterial antigens may activate T-cell and inflammation by antigen mimicry mechanism [47–49]. Finally, the most recent theory is based on the endoplasmic reticulum stress: under normal conditions, the peptide-loaded HLA class I heavy chain binds the *β*2-microglobulin (*β*2m) in the endoplasmic reticulum. The folding process of the HLA-B27 heavy chain is slower than that of other HLA alleles thus leading to the generation of misfolded chains. Misfolded chains are usually removed in the endoplasmic reticulum, but in certain conditions, such as viral infection, they accumulate thus activating the protein BiP, the endoplasmic reticulum-unfolded-protein-response (UPR) and the nuclear factor *κ*B (NF*κ*B), which play a critical role in the induction of inflammation [50]. Data suggest that deposition of *β*2m, caused by the high dissociation rate between HLA-B27 heavy chain and *β*2m, occurring within synovial tissue, may lead to the initiation of chronic inflammation [51, 52].

Other HLA genes have been associated with SpA in IBD: HLA-DrB10103, HLA-B35, HLA-B24 in type 1 peripheral arthritis, and HLA-B44 in type 2 peripheral arthritis [43]. Moreover, Mielants et al. showed an association between HLA-Bw62 and chronic gut lesions associated with a family history of AS and CD, with markers of inflammation, reduced axial mobility, presence of sacroiliitis, destructive joint lesions, and a diagnosis of AS [53].

Further confirming the relevance of HLA-B27 in the pathogenesis of enteroarthritis, Hammer et al. studied transgenic rats overexpressing HLA-B27 molecule. These rats developed a multisystemic inflammatory disease that had several clinical and histopathological similarities to SpA and IBD [54]. An important finding was that these rats did not develop joint or gut inflammation when they were in a germ-free environment [55]. This result supports the theory of the participation of microorganisms in the pathogenesis of these diseases. 

In humans with reactive arthritis, following Yersinia enterocolitica, Shigella spp. or Salmonella enteritidis and typhimurium infection, bacterial antigens have been detected in joints [56–59]. Later studies further confirmed this evidence [60–62].Other molecular studies found similarities between Klebsiella nitrogenase and HLA-B27 and between Klebsiella pullulanase and collagen fibers types I, III, and IV. Interestingly, elevated levels of antibodies against Klebsiella and collagen fibers types I, III, IV, and V were detected in patients with CD and AS [63]. 

In addition to HLA-B27, other genes have been identified as being related to SpA and IBD. In fact, several common genetic predispositions between SpA and IBD were identified, of which the association with IL-23R polymorphisms is most prominent [64]. The functional role of IL-23 receptor polymorphisms remains unclear, the fact that IL-23 signaling plays a critical role in the Th17-mediated inflammation indicates that Th17 cells may represent a common pathogenetic mechanism in both IBD and SpA [65]. The first susceptibility gene that has been identified for CD is CARD15 (or NOD2). Variants within this gene increase the risk for CD by threefold for heterozygous and fortyfold for homozygous individuals. An association was also found in SpA patients between the carriage of CARD15 variants and the development of chronic subclinical gut inflammation [66]. Although CARD15 mutations do not seem to predispose to arthritis, it might confer a risk towards the development of (sub) clinical gut inflammation in SpA patients, rendering these patients more disposed to develop IBD. A CARD15-mediated NF*κ*B-dependent inflammatory reaction might be an important pathogenic process within the joints [67]. The protein is expressed in joint tissue, and bacterial cell wall components have been demonstrated in synovium of SpA patients, supporting the idea that CARD15 can locally trigger inflammation [68]. Recently, additional shared associations between SpA and IBD were found at chromosome 1q32 near KIF21B (genome-wide significant), STAT3, IL-12B, CDKAL1, LRRK2/MUC19, and chromosome 13q14 (experiment-wise association). As the genes IL-23R, STAT3, and IL-12B all influence Th17 lymphocyte differentiation/activation, this provides further evidence implicating the Th17 lymphocyte subset in the pathogenesis of SpA [65].

**Figure 1:**
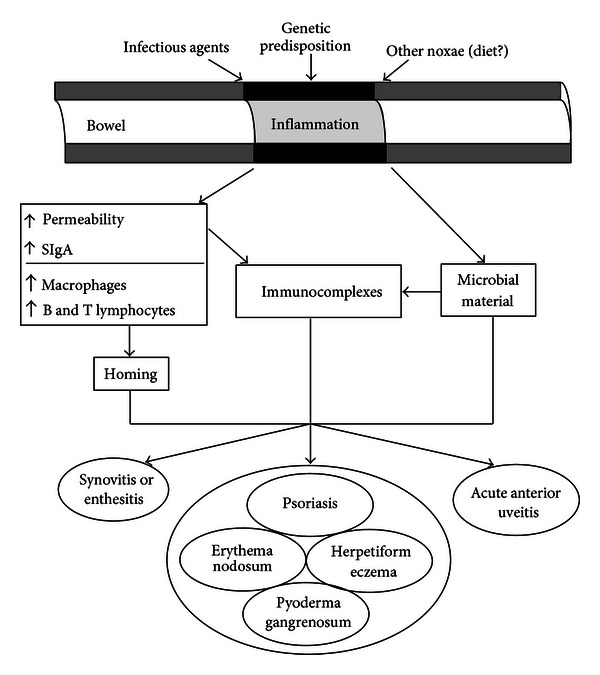
The bowel component in the pathogenesis of seronegative spondyloarthritis.

In addition to genetic susceptibility, an important role was also been given to the environmental factors in triggering the onset of disease. In fact, bacterial gut infections such as Yersinia enterocolitica, Salmonella typhimurium, Campylobacter jejuni, and Shigella spp may cause joint inflammation in genetically predisposed patients. Given the prototypical link between certain bacterial infections and the onset of reactive arthritis, several studies have aimed to assess the role of intestinal flora in disease progression, as well as the resulting changes in mucosal response [69]. On the basis of these observation, the possible pathways involved in joint and gut inflammation in EA may be the following: in the acute phase of inflammation, bacterial infections can cause acute intestinal inflammation. Certain bacteria may survive intracellularly in macrophages that can traffic to the joint and cause an arthritis in a genetically predisposed host. Proinflammatory cytokines such as TNF and IL-23 are produced locally, with Paneth cells being the most important producers of IL-23 in the intestine. This expression can activate innate immune cells (NK) to produce IL-22 that may help control inflammation at mucosal sites. Otherwise, damage and pathogen associated molecular pattern molecules (DAMPs and PAMPs) and cellular stretch might promote initiation of joint inflammation. In the transition phase, acute intestinal and articular inflammation can be sustained due to defective immune regulation by TREG cells, or by ER stress, whereas iNKT cells act as regulators to control inflammation. Proangiogenic factors such as PlGF can lead to aberrant neovascularisation. These events may lead to chronicity, further enhanced or maintained by repetitive cellular stress. In this stage, stromal cells become more important, as targets for proinflammatory cytokines (Figure 1).

### 4.1. Clinical Pattern

The joint involvement observed in IBD is classified in two subsets: peripheral and axial (including sacroiliitis with or without spondylitis) [24]. There may be other periarticular manifestations such as enthesopathy, dactylitis, tendonitis, periostitis, clubbing, granulomatous lesions (in joints and bones), osteoporosis, and osteomalacia [70]. 

The arthritic manifestations of IBD are divided into different clinical subsets: peripheral and axial joint involvement (including sacroiliitis with or without spondylitis). Peripheral arthritis is the most frequent finding in both CD and UC and may occur with a frequency ranging between 17% and 20% [24], and it is more common in CD [71]. The peripheral arthritis equally affects both the sexes and the onset age is between 25 and 45 years. It can diagnosed before, simultaneously, or after the diagnosis of IBD. It is generally acute and it reaches the symptoms apex within 48 hours. The clinical picture is that of an asymmetric monooligoarthritis which affects especially the large joints of the lower limbs, less frequently those of the upper limbs. In general, the small joints involvement is more typical of CD. The course is episodic and recurrent, with spontaneous reduction of symptoms within about 6 months; a small percentage tends to become chronic. In addition, exacerbations are characterizing, connected with the activity stages of the inflammatory bowel disease. The nature of arthritis is generally related to the extension and severity of intestinal involvement, a more evident aspect of the UC, and to the incidence of extraintestinal complications, such as uveitis, stomatitis, erythema nodosum, and pyoderma gangrenosum. A stable reduction of arthritis is often observed after a colectomy. In 1998, Orchard et al. [26] distinguished two subtypes of peripheral arthritis: type 1, the pauciarticular form (involving fewer than 5 joints), acute and self-limited, which may precede the diagnosis of IBD, generally running parallel to the intestinal disease; type 2, the polyarticular form (involving 5 or more joints), with symptoms lasting for months or years, independently from inflammatory bowel disease. More recently, Smale et al. have shown another type of peripheral arthritis (type 3) that includes patients with both axial and peripheral forms [72]. 

The axial involvement is more common in patients with CD (5%–22%) than in those with UC (2%–6%) [19]. Its onset precedes the enteritis and its course is not related to it [73]. Both progressive ankylosing spondylitis and sacroiliitis (sometimes asymptomatic) may occur. Ankylosing spondylitis (AS) affects the vertebral column by progressive ankylosis of the vertebral and the sacroiliac joints. The clinical course of AS in IBD is similar to idiopathic AS, and disease progression leads to increasing immobility of the spine resulting in ankylosis (bamboo spine) [73]. 

Radiological examination of the affected joints basically shows the absence of erosions as well as of osteoporosis or joint narrowing. The use of joint scintigraphy is helpful, as, in some cases, this technique shows the hyper accumulation of the tracer even in presence of negative radiological findings, particularly on a sacroiliac level. Recently, some studies have revealed that the axial subset incidence is higher than the previously reported one, as formerly described by our research team in 1992 [21]. In fact, in these studies, based on the ESSG SpA criteria [17], the authors have detected a prevalence between 10% and 25% of spondylitis, and between 30% and 36% of sacroiliitis [20, 22, 23]. A complication of the axial subset in patients with IBD is the development of sclerotic erosive lesions in the adjacent vertebral bodies [74] or aseptic spondylodiscitis, also defined as Andersson lesions (ALs) [75], whose diagnosis is often delayed, particularly in patients having an insidious onset and nonspecific symptoms [76]. Our research group has recently investigated the occurrence and the clinical features of ALs arthritis in EA, detected by magnetic resonance imaging, an imaging technique usually accepted to evaluate the axial and peripheral involvement of SpA [77–79], and we found an high prevalence of this lesion among EA patients (30.55%), confirming that ALs are an important characteristic aspect of SpA. In particular, we found a high prevalence in patients with axial and peripheral subset (31.82%) suggesting that ALs could be a characterizing feature of the overlap subset [57]. Furthermore, in contrast with what was reported for ankylosing spondylitis [80] and psoriatic arthritis [81], the occurrence of these lesions in patients with UC and CD was earlier and often asymptomatic [82] (Table 1). 

## 5. Other Rheumatic and Extra-Articular Manifestations

IBD is associated, less frequently, with other rheumatic disease such as rheumatoid arthritis [83], Sjogren syndrome [84], Takayasu arteritis [85, 86], and fibromyalgia [87].

Enthesitis, dactylitis, and buttock pain are a part of EA and their occurrences are not different from the other SpA.

The extra-articular manifestations are characterized by acute anterior uveitis, aortic insufficiency, and cardiac conduction disturbances with a frequency of 25%, 4%–10%, and 3%–9%, respectively. They seem to be related to disease duration, axial joint involvement, and with HLA-B27 positivity. Skin lesions occur in 10%–25% of the patients and are characterized by erythema nodosum, coinciding with exacerbations of the gut inflammation and thus tends to occur in patients with active peripheral synovitis [26], and pyoderma gangrenosum, not related to gut inflammation, which is reported in 2.2% of UC patients and 1.5% of CD patients with a female predominance [88, 89] and is considered to be the most severe skin manifestation in IBD. 

Recently, our research group has investigated the occurrence of chronic autoimmune thyroiditis or Hashimoto's thyroiditis (HT) in EA patients [90]. HT is known to be an extraintestinal complications of IBD and the increased prevalence of thyroid antibodies in ulcerative colitis patients has been reported to range from 0.82% [91] to 3.7% [92]. Our results show that HT occurs more frequently in EA patients. In details, TPOAbs positivity occurs more frequently in patients with a long disease duration and active rheumatic disease than in the other patients suggesting a possible relationship between the maintenance of the inflammatory process in EA patients and the positivity of TPOAbs. Furthermore EA patients affected by thyroiditis show a peripheral involvement, with a significantly high prevalence of polyarticular joint involvement [90].

## 6. Cardiovascular Disease and EA

Several literature data clearly suggest that subjects with IBD, as well as those with spondyloarthritides, show an increased thrombotic risk. Significantly less data are available about EA subjects. 

Both venous and arterial thrombosis risks have been studied in IBD subjects. Many reports document an increased risk of venous thrombotic events in patients with IBD while the risk of arterial thrombotic events has been less well characterized [93]. However, some studies described an increased cardiovascular (CV) risk in this clinical setting [94]. Common carotid artery intima-media thickness (IMT) is significantly higher in IBD subjects as compared with controls [95]; so IBD seems to be a risk factor for cardiovascular and cerebrovascular events [96–99] even in young patients. Premature subclinical atherosclerosis with high-density lipoprotein cholesterol values, high IMT, and reduced flow-mediated dilation (FMD) has been reported in children with inflammatory bowel disease [100]. Moreover, a frequent occurrence of metabolic syndrome (MS) was documented in IBD, especially in UC [101]. Also the risk of hyperhomocysteinaemia is significantly higher in IBD patients when compared with controls [102–104], and an increased prevalence of hepatic steatosis, which is a recognized predictor of arterial and venous thrombosis, has been found both in the IBD population [105] and in subjects with enteropathic arthritis [106, 107]. 

Female gender seems to further increase CV risk among IBD patients [108]. A retrospective cohort study revealed that women over the age of 40 years with IBD are at increased risk of myocardial infarction. In contrast, this report showed that men with IBD do not share the same risks for arterial thrombotic events. The study also revealed that IBD patients have a significant risk of acute mesenteric ischemia when compared with controls [93]. Accordingly, another study explored comorbidities in IBD patients, showing an increased prevalence of coronary artery disease (OR 1.883, *P* = 0.001) with a higher risk in female patients (OR 1.6, *P* = 0.014) [108].

Whereas many studies are consistent about the increased risk of arterial thrombotic events, some studies have failed to demonstrate an increased CV risk in IBD subjects [109, 110]. Interestingly, the retrospective study by Ha et al. [93] did not find an increased risk of coronary artery disease (CAD) in IBD subjects but showed a high prevalence of hypertension and hyperlipidemia. Conversely, another retrospective study showed that IBD patients had an increased risk of CAD but lower rates of the traditional risk factors for CAD (obesity, diabetes mellitus, hypertension, and hyperlipidemia) [94]. Furthermore, a recent meta-analysis [111] (including 11 studies for a total of 14.065 patients) was not able to demonstrate an increased cardiovascular mortality in the IBD population. Anyway, the authors themselves found some limitations that can explicate these results, such as the younger age, the short duration of followup, the protective role of lower BMI and lipid levels, or lower cigarette use of these patients. Thus, further properly designed studies are needed to address these issues. 

An increased CV risk has been documented also in subjects with spondyloarthritides [112, 113] and other systemic inflammatory diseases, such as rheumatoid arthritis, Sjogren syndrome, and systemic lupus erythematosus [114, 115]. Both in psoriatic arthritis and in AS, an impaired vascular flow-mediated dilation and carotid intima-media thickening [116–118] have been found. In AS the coronary flow reserve and the left ventricular diastolic function are impaired and these findings are positively correlated with inflammatory indices, such as C-reactive protein [119]. However, other studies do not confirm the finding of an increased CV risk in AS subjects [120, 121].

Despite the common inflammatory pathogenesis and the evidences of increased CV risk both in IBD and in SpA, there are no literature data on the CV risk in EA patients.

In addition, according to recent data [122] suggesting a strict correlation between arterial and venous thrombosis, it is interesting to highlight that a series of studies are consistent with the hypothesis of an increased venous thrombosis risk in IBD subjects [93, 123–125]. Patients with IBD have an increased risk of venous thrombotic events. Recently, a history of Venous Thrombo Embolism (VTE) has been documented in 22.5% of IBD patients, who reported a total of 21 thromboembolic manifestations, including 15 deep venous thrombosis (DVT), 3 thrombophlebitis, 2 thrombosis of the superficial femoral artery, and 1 thrombosis of the central retinal vein. A disregulation of the haemostatic system was also described, regardless the occurrence of thromboembolic events. Thrombocytosis was observed in 33% of IBD patients, hyperhomocysteinemia in 26.7%, increased D-dimer values in 25.3%, elevated C3 in 15.4%, and resistance to activated protein C (APCr) in 5.6% [93]. This study further confirms a thromboembolic tendency in IBD patients and also suggests the occurrence of a disregulation of the hemostatic system. 

Many other reports show an hemostatic disequilibrium, mainly in the active form of IBD, documenting higher levels of procoagulant factors (XI, XII, X, V), prothrombin and side-products of prothrombin cleavage and, on the other hand, lower or normal levels of anticoagulant factors (protein S, protein C, and antithrombin III) and a reduced activity of the fibrinolytic system [126–131]. Also endothelial dysfunction with impaired NO production has been found in IBD subjects [132, 133]. From 1968 [134] the association between thrombocytosis and IBD is known and later studies found changes in platelet count [103], volume [135] and activation [136] and an increased number of circulating platelet-leukocyte aggregates [137]. Finally, a recent study found that CD and UC patients also have an increased risk of developing pericarditis compared to healthy controls [138].

Overall, given changes in the haemostatic balance, as well as the evidence of an increased IMT both in IBD and in SpA subjects, the evaluation of the CV risk in EA subjects is now mandatory. 

## 7. Treatment

The management of patients with EA requires an active cooperation between gastroenterologist and rheumatologist. 

The use of corticosteroids and/or DMARDs and/or of anti-TNF*α*, helpful to contain intestinal inflammation, usually leads also to the reduction of peripheral type I arthritis symptoms, which also well respond to rest, to physiokinesitherapy and to intra-articular injections of steroids. On the contrary, the management of types II and III is more complex and they may persist despite the reduction of IBD. Our experience suggests that the majority of patients promptly respond to anti-inflammatory drugs, useful to control joint and entheses inflammation. However, they do not stop the development of joint damage and, at the same time, they may also be responsible for important side effects on the bowel, such as the exacerbation of IBD [139, 140], thus causing the appearance of small intestine and colon ulcers [141]. As a consequence, in order to manage joint symptoms, these drugs are recommended for patients with mild exacerbations but their use should be limited to the lowest effective dose and only for short periods of time. 

Sulfasalazine and 5-aminosalicylic acid are often used for the treatment of IBD, being their effectiveness also confirmed for the management of mild peripheral arthritis, particularly in patients with UC [142, 143]. Their effectiveness on CD has not been well proved yet. These drugs have no effect on the evolution of joint damage to severe forms of arthritis and their usefulness in the axial subset is marginal; they do not seem to prevent the possible onset of intestinal inflammation in patients with SpA [144]. 

Immunosuppressants such as methotrexate, azathioprine, cyclosporine, and leflunomide show their efficacy in some patients with peripheral arthritis and other extraintestinal components [145–149]. Recently, our team has been studying the efficacy and the tolerability of methotrexate at a dose of 20 mg/week, in patients with peripheral arthritis under UC, and we have shown a rapid and effective reduction of joint symptoms with significant improvement in laboratory parameters and in rates of disease activity [150]. 

The anti-TNF*α*, especially infliximab and adalimumab, are showed to be successful in the restraint not only of the intestinal inflammation but also of the joint clinical picture (axial and peripheral), especially in patients with CD; currently, these drugs are widely used to treat enteric arthritis [151]. Etanercept, instead, seems to be effective only to control joint symptoms but not the intestinal ones [152, 153]. 

Some authors [154–156] have also proposed to use probiotics in order to treat patients with inflammatory bowel disease and arthritis. Probiotics alter intestinal flora and limit subjects' persistent arthralgias in the early stages of the disease, that is, before joint damage onset, thus improving the quality of life and positively affecting the natural course of the disease. Some studies have also shown a possible improvement of experimental colitis in mouse models and in patients with inflammatory bowel disease [157]. One of these models has shown that the anti-inflammatory effect depends on the probiotic DNA, in an IFN-mediated response induced by the Toll-like receptor 9 (TLR9), a specific receptor for nonmethylated DNA sequences (CpG motifs), mainly shown by bacteria, which are able to modulate the immune response [158].

Finally, in consideration of the high CV risk among IBD patients, common cardiovascular drugs (statins and angiotensin-converting enzyme inhibitors) may have dual potential for preventing or treating coronary artery disease and controlling inflammatory bowel disease [159].

## Figures and Tables

**Table 1:** Classification and features of articular involvement subsets in inflammatory bowel disease (IBD).

Peripheral			Axial	
Type 1	Type 2	Type 3	Isolated sacroiliitis	Spondylitis
(i) Pauciarticular (less than 5 joints) (ii) Asymmetric involvement (iii) Acute, self-limiting attack (<10 weeks) (iv) Usually coincides with relapse of IBD (v) Strongly associated with other extra-intestinal manifestations (vi) Lower limbs more affected (vii) Associated with HLA DRB1, B35, B27	(i) Polyarticular (5 or more joints) (ii) Symptoms persist for months or even years (iii) May be erosive (iv) Runs a course independent of IBD (v) Affects both large and small joints (vi) Strongly associated with uveitis (vii) Associated with HLA B44	(i) Both axial and peripheral involvement	(i) Asymptomatic (ii) Usually non progressive disease	(i) Usually precede the onset of IBD (ii) Runs a course independent of IBD (iii) Clinical course is similar to idiopathic ankylosing spondylitis (iv) Disease progression leads to increasing immobility and ankylosing (v) Associated with uveitis (vi) Strongly associated with HLA B27

## References

[1] Leirisalo-Repo M (1994). Enteropathic arthritis, Whipple’s disease, juvenile spondyloarthropathy, and uveitis. *Current Opinion in Rheumatology*.

[2] Orlando A, Renna S, Perricone G, Cottone M (2009). Gastrointestinal lesions associated with spondyloarthropathies. *World Journal of Gastroenterology*.

[3] Smith R (1922). Treatment of rheumatoid arthritis by colectomy. *Annals of Surgery*.

[4] Bargen JA, Jackman RJ, Kerr JG (1929). Complications and sequel of chronic ulcerative colitis. *Annals of Internal Medicine*.

[5] Hench PS, Whipple GH (1935). Acute and chronic arthritis. *Nelson’s Loose Leaf of Surgery*.

[6] Bywaters EG, Ansell BM (1958). Arthritis associated with ulcerative colitis; a clinical and pathological study. *Annals of the Rheumatic Diseases*.

[7] McBride JA, King MJ, Baikie AG, Crean GP, Sircus W (1963). Ankylosing spondylitis and chronic inflammatory diseases of the intestines. *British Medical Journal*.

[8] Steinberg VL, Storey G (1957). Ankylosing spondylitis and chronic inflammatory lesions of the intestines. *British Medical Journal*.

[9] Stewart JS, Ansell BM (1963). Ankylosing spondylitis associated with regional enteritis. *Gastroenterology*.

[10] Blumberg BS, Bunim JJ, Calkins E, Pirani CL, Zvaifler NJ (1964). Aranomenclature and classification of arthritis and rheumatism (tentative). *Arthritis and Rheumatism*.

[11] Wright V, Moll JHM (1976). *Seronegative Polyarthritis*.

[12] Puite RH, Tesluk H (1955). Whipple’s disease. *The American Journal of Medicine*.

[13] Kelly JJ, Weisiger BB (1963). The arthritis of Whipple's disease. *Arthritis and Rheumatism*.

[14] Friis J, Svejgaard A (1974). Letter: Salmonella arthritis and HL-A27. *The Lancet*.

[15] Aho K, Ahvonen P, Lassus A, Dumond DCP (1976). Yersinia arthritis and related diseases. *Infection and Immunology in the Rheumatic Diseases*.

[16] Braun J, Sieper J (2002). Building consensus on nomenclature and disease classification for ankylosing spondylitis: results and discussion of a questionnaire prepared for the International Workshop on New Treatment Strategies in Ankylosing Spondylitis, Berlin, Germany, 18-19 January 2002. *Annals of the Rheumatic Diseases*.

[17] Dougados M, van der Linden S, Juhlin R (1991). The European Spondylarthropathy Study Group preliminary criteria for the classification of spondylarthropathy. *Arthritis and Rheumatism*.

[18] Cosnes J, Gower-Rousseau C, Seksik P, Cortot A (2011). Epidemiology and natural history of inflammatory bowel diseases. *Gastroenterology*.

[19] Salvarani C, Fries W (2009). Clinical features and epidemiology of spondyloarthritides associated with inflammatory bowel disease. *World Journal of Gastroenterology*.

[20] Turkcapar N, Toruner M, Soykan I (2006). The prevalence of extraintestinal manifestations and HLA association in patients with inflammatory bowel disease. *Rheumatology International*.

[21] Scarpa R, del Puente A, D’Arienzo A (1992). The arthritis of ulcerative colitis: clinical and genetic aspects. *Journal of Rheumatology*.

[22] de Vlam K, Mielants H, Cuvelier C, de Keyser F, Veys EM, de Vos M (2000). Spondyloarthropathy is underestimated in inflammatory bowel disease: prevalence and HLA association. *Journal of Rheumatology*.

[23] Wordsworth P (2000). Arthritis and inflammatory bowel disease. *Current Rheumatology Reports*.

[24] Rodríguez-Reyna TS, Martínez-Reyes C, Yamamoto-Furusho JK (2009). Rheumatic manifestations of inflammatory bowel disease. *World Journal of Gastroenterology*.

[25] Palm O, Moum B, Jahnsen J, Gran JT (2001). The prevalence and incidence of peripheral arthritis in patients with inflammatory bowel disease, a prospective population-based study (the IBSEN study). *Rheumatology*.

[26] Orchard TR, Wordsworth BP, Jewell DP (1998). Peripheral arthropathies in inflammatory bowel disease: their articular distribution and natural history. *Gut*.

[27] Yüksel I, Ataseven H, Başar O (2011). Peripheral arthritis in the course of inflammatory bowel diseases. *Digestive Diseases and Sciences*.

[28] Vavricka SR, Brun L, Ballabeni P (2011). Frequency and risk factors for extraintestinal manifestations in the swiss inflammatory bowel disease cohort. *American Journal of Gastroenterology*.

[29] Veloso FT, Carvalho J, Magro F (1996). Immune-related systemic manifestations of inflammatory bowel disease: a prospective study of 792 patients. *Journal of Clinical Gastroenterology*.

[30] Manguso F, Staiano T, Astarita C (2005). Consecutive occurrence of rhinoconjunctivitis, seronegative spondyloarthritis and pyoderma gangrenosum in a patient with ulcerative colitis. *International Journal of Colorectal Disease*.

[31] Manguso F, Sanges M, Staiano T (2004). Cigarette smoking and appendectomy are risk factors for extraintestinal manifestations in ulcerative colitis. *American Journal of Gastroenterology*.

[32] Cuvelier C, Barbatis C, Mielants H, de Vos M, Roels H, Veys E (1987). Histopathology of intestinal inflammation related to reactive arthritis. *Gut*.

[33] de Vos M, Cuvelier C, Mielants H, Veys E, Barbier F, Elewaut A (1989). Ileocolonoscopy in seronegative spondylarthropathy. *Gastroenterology*.

[34] Mielants H, Veys EM, Joos R, Cuvelier C, de Vos M (1987). Repeat ileocolonoscopy in reactive arthritis. *Journal of Rheumatology*.

[35] Mielants H, Veys EM, Cuvelier C, de Vos M, Botelberghe L (1985). HLA-B27 related arthritis and bowel inflammation—part 2: ileocolonoscopy and bowel histology in patients with HLA-B27 related arthritis. *Journal of Rheumatology*.

[36] Scarpa R, Manguso F, D’Arienzo A (2000). Microscopic inflammatory changes in colon of patients with both active psoriasis and psoriatic arthritis without bowel symptoms. *Journal of Rheumatology*.

[37] Jacques P, Elewaut D, Mielants H (2010). Interactions between gut inflammation and arthritis/spondylitis. *Current Opinion in Rheumatology*.

[38] Jacques P, Elewaut D (2008). Joint expedition: linking gut inflammation to arthritis. *Mucosal Immunology*.

[39] Schlosstein L, Terasaki PI, Bluestone R, Pearson CM (1973). High association of an HL-A antigen, W27, with ankylosing spondylitis. *The New England Journal of Medicine*.

[40] Brown MA, Pile KD, Kennedy LG (1996). HLA class I associations of ankylosing spondylitis in the white population in the United Kingdom. *Annals of the Rheumatic Diseases*.

[41] Mallas EG, Mackintosh P, Asquith P, Cooke WT (1976). Histocompatibility antigens in inflammatory bowel disease; their clinical significance and their association with arthropathy with special reference to HLA B27 (W27). *Gut*.

[42] Peeters H, Vander Cruyssen B, Mielants H (2008). Clinical and genetic factors associated with sacroiliitis in Crohn’s disease. *Journal of Gastroenterology and Hepatology*.

[43] Orchard TR, Thiyagaraja S, Welsh KI, Wordsworth BP, Gaston JSH, Jewell DP (2000). Clinical phenotype is related to HLA genotype in the peripheral arthropathies of inflammatory bowel disease. *Gastroenterology*.

[44] Kuon W, Sieper J (2003). Identification of HLA-B27-restricted peptides in reactive arthritis and other spondyloarthropathies: computer algorithms and fluorescent activated cell sorting analysis as tools for hunting of HLA-B27-restricted chlamydial and autologous crossreactive peptides involved in reactive arthritis and ankylosing spondylitis. *Rheumatic Disease Clinics of North America*.

[45] Mertz AKH, Wu P, Sturniolo T (2000). Multispecific CD4^+^ T cell response to a single 12-mer epitope of the immunodominant heat-shock protein 60 of Yersinia enterocolitica in Yersinia- triggered reactive arthritis: overlap with the B27-restricted CD8 epitope, functional properties, and epitope presentation by multiple DR alleles. *Journal of Immunology*.

[46] Thiel A, Wu P, Lauster R, Braun J, Radbruch A, Sieper J (2000). Analysis of the antigen-specific T cell response in reactive arthritis by flow cytometry. *Arthritis & Rheumatism*.

[47] Scofield RH, Kurien B, Gross T, Warren WL, Harley JB (1995). HLA-B27 binding of peptide from its own sequence and similar peptides from bacteria: implications for spondyloarthropathies. *The Lancet*.

[48] Ramos M, Alvarez I, Sesma L, Logean A, Rognan D, de López Castro JA (2002). Molecular mimicry of an HLA-B27-derived ligand of arthritis-linked subtypes with chlamydial proteins. *Journal of Biological Chemistry*.

[49] Frauendorf E, von Goessel H, May E, Märker-Hermann E (2003). HLA-B27-restricted T cells from patients with ankylosing spondylitis recognize peptides from B*2705 that are similar to bacteria-derived peptides. *Clinical & Experimental Immunology*.

[50] Fantini MC, Pallone F, Monteleone G (2009). Common immunologic mechanisms in inflammatory bowel disease and spondylarthropathies. *World Journal of Gastroenterology*.

[51] Baeten D, de Keyser F, Mielants H, Veys EM (2002). Immune linkages between inflammatory bowel disease and spondyloarthropathies. *Current Opinion in Rheumatology*.

[52] Uchanska-Ziegler B, Ziegler A (2003). Ankylosing spondylitis: a *β*2m-deposition disease?. *Trends in Immunology*.

[53] Mielants H, Veys EM, Goemaere S, Goethals K, Cuvelier C, de Vos M (1991). Gut inflammation in the spondyloarthropathies: clinical, radiologic, biologic and genetic features in relation to the type of histology. A prospective study. *Journal of Rheumatology*.

[54] Hammer RE, Maika SD, Richardson JA, Tang JP, Taurog JD (1990). Spontaneous inflammatory disease in transgenic rats expressing HLA-B27 and human *β*2m: an animal model of HLA-B27-associated human disorders. *Cell*.

[55] Taurog JD, Richardson JA, Croft JT (1994). The germfree state prevents development of gut and joint inflammatory disease in HLA-B27 transgenic rats. *Journal of Experimental Medicine*.

[56] Hammer M, Zeidler H, Klimsa S, Heesemann J (1990). Yersinia enterocolitica in the synovial membrane of patients with Yersinia-induced arthritis. *Arthritis and Rheumatism*.

[57] Granfors K, Jalkanen S, von Essen R (1989). Yersinia antigens in synovial-fluid cells from patients with reactive arthritis. *The New England Journal of Medicine*.

[58] Granfors K, Jalkanen S, Toivanen P, Koski J, Lindberg AA (1992). Bacterial lipopolysaccharide in synovial fluid cells in Shigella triggered reactive arthritis. *Journal of Rheumatology*.

[59] Granfors K, Jalkanen S, Lindberg AA (1990). Salmonella lipopolysaccharide in synovial cells from patients with reactive arthritis. *The Lancet*.

[60] Kirveskari J, Jalkanen S, Mäki-Ikola O, Granfors K (1998). Increased synovial endothelium binding and transendothelial migration of mononuclear cells during Salmonella infection. *Arthritis & Rheumatism*.

[61] Wuorela M, Tohka S, Granfors K, Jalkanen S (1999). Monocytes that have ingested Yersinia enterocolitica serotype O:3 acquire enhanced capacity to bind to nonstimulated vascular endothelial cells via P-selectin. *Infection and Immunity*.

[62] Wuorela M, Jalkanen S, Kirveskari J, Laitio P, Granfors K (1997). Yersinia enterocolitica serotype O:3 alters the expression of serologic HLA-B27 epitopes on human monocytes. *Infection and Immunity*.

[63] Ebringer A, Rashid T, Tiwana H, Wilson C (2007). A possible link between Crohn’s disease and ankylosing spondylitis via Klebsiella infections. *Clinical Rheumatology*.

[64] Duerr RH, Taylor KD, Brant SR (2006). A genome-wide association study identifies IL23R as an inflammatory bowel disease gene. *Science*.

[65] van Praet L, van den Bosch F, Mielants H, Elewaut D (2011). Mucosal inflammation in spondylarthritides: past, present, and future. *Current Rheumatology Reports*.

[66] Laukens D, Peeters H, Marichal D (2005). CARD15 gene polymorphisms in patients with spondyloarthropathies identify a specific phenotype previously related to Crohn’s disease. *Annals of the Rheumatic Diseases*.

[67] Miceli-Richard C, Zouali H, Lesage S (2002). CARD15/NOD2 analyses in spondylarthropathy. *Arthritis and Rheumatism*.

[68] de Vos M, Hindryckx P, Laukens D (2011). Novel development in extraintestinal manifestations and spondylarthropathy. *Best Practice and Research: Clinical Gastroenterology*.

[69] van Praet L, Jacques P, van den Bosch F, Elewaut D (2012). The transition of acute to chronic bowel inflammation in spondyloarthritis. *Nature Reviews Rheumatology*.

[70] Holden W, Orchard T, Wordsworth P (2003). Enteropathic arthritis. *Rheumatic Disease Clinics of North America*.

[71] Gravallese EM, Kantrowitz FG (1988). Arthritic manifestations of inflammatory bowel disease. *American Journal of Gastroenterology*.

[72] Smale S, Natt RS, Orchard TR, Russell AS, Bjarnason I (2001). Inflammatory bowel disease and spondylarthropathy. *Arthritis & Rheumatism*.

[73] Rothfuss KS, Stange EF, Herrlinger KR (2006). Extraintestinal manifestations and complications in inflammatory bowel diseases. *World Journal of Gastroenterology*.

[74] Calin A, Robertson D (1991). Spondylodiscitis and pseudarthrosis in a patient with enteropathic spondyloarthropathy. *Annals of the Rheumatic Diseases*.

[75] Andersson O (1937). Röntgenbilden vid spondylarthritis ankylopoetica. *Nordisk Medicinsk Tidskrift*.

[76] Zöld E, Barta Z, Zeher M (2007). Spondylodiscitis representing as the very first sign of Crohn’s disease. *Inflammatory Bowel Diseases*.

[77] Lambert RGW, Pedersen SJ, Maksymowych WP, Chiowchanwisawakit P, Østergaard M (2009). Active inflammatory lesions detected by magnetic resonance imaging in the spine of patients with spondyloarthritis—definitions, assessment system, and reference image set. *Journal of Rheumatology*.

[78] Østergaard M, Maksymowych WP, Pedersen SJ, Chiowchanwisawakit P, Lambert RGW (2009). Structural lesions detected by magnetic resonance imaging in the spine of patients with spondyloarthritis—definitions, assessment system, and reference image set. *Journal of Rheumatology*.

[79] Soscia E, Scarpa R, Cimmino MA (2009). Magnetic resonance imaging of nail unit in psoriatic arthritis. *Journal of Rheumatology. Supplement*.

[80] Kabasakal Y, Garrett SL, Calin A (1996). The epidemiology of spondylodiscitis in ankylosing spondylitis. A controlled study. *British Journal of Rheumatology*.

[81] Scarpa R (2000). Discovertebral erosions and destruction in psoriatic arthritis. *Journal of Rheumatology*.

[82] Peluso R, Di Minno MN, Bruner V (2012). Discovertebral erosions in patients with enteropathic spondyloarthritis. *The Journal of Rheumatology*.

[83] Toussirot E, Wendling D (1997). Crohn’s disease associated with seropositive rheumatoid arthritis. *Clinical and Experimental Rheumatology*.

[84] Rhew EY, Ramsey-Goldman R, Buchman AL (2003). Sjogren’s syndrome in association with Crohn’s disease. *Journal of Clinical Gastroenterology*.

[85] Aoyagi S, Akashi H, Kawara T (1998). Aortic root replacement for Takayasu arteritis associated with ulcerative colitis and ankylosing spondylitis: report of a case. *Japanese Circulation Journal*.

[86] Hilário MOE, Terreri MTRA, Prismich G (1998). Association of anky-losing spondylitis, Crohn’s disease and Takayasu’s arteritis in a child. *Clinical and Experimental Rheumatology*.

[87] Palm O, Moum B, Jahnsen J, Gran JT (2001). Fibromyalgia and chronic widespread pain in patients with inflammatory bowel disease: a cross sectional population survey. *Journal of Rheumatology*.

[88] Bernstein CN, Blanchard JF, Rawsthorne P, Yu N (2001). The prevalence of extraintestinal diseases in inflammatory bowel disease: a population-based study. *American Journal of Gastroenterology*.

[89] Yüksel I, Başar O, Ataseven H (2009). Mucocutaneous manifestations in inflammatory bowel disease. *Inflammatory Bowel Diseases*.

[90] Peluso R, Lupoli GA, Del Puente A (2011). Prevalence of thyroid autoimmunity in patients with spondyloarthropathies. *Journal of Rheumatology*.

[91] Triantafillidis JK, Manoussakis CA, Tsafaras C, Koutsorizof A (1990). Coexistence of thyreotoxicosis and exacerbation of ulcerative colitis. *American Journal of Gastroenterology*.

[92] Järnerot G, Azad Khan AK, Truelove SC (1975). The thyroid in ulcerative colitis and Crohn’s disease. II. Thyroid enlargement and hyperthyroidism in ulcerative colitis. *Acta Medica Scandinavica*.

[93] Ha C, Magowan S, Accortt NA, Chen J, Stone CD (2009). Risk of arterial thrombotic events in inflammatory bowel disease. *American Journal of Gastroenterology*.

[94] Yarur AJ, Deshpande AR, Pechman DM, Tamariz L, Abreu MT, Sussman DA (2011). Inflammatory bowel disease is associated with an increased incidence of cardiovascular events. *The American Journal of Gastroenterology*.

[95] Papa A, Santoliquido A, Danese S (2005). Increased carotid intima-media thickness in patients with inflammatory bowel disease. *Alimentary Pharmacology and Therapeutics*.

[96] Efremidis M, Prappa E, Kardaras F (1999). Acute myocardial infarction in a young patient during an exacerbation of ulcerative colitis. *International Journal of Cardiology*.

[97] Mutlu B, Ermeydan CMH, Enç F (2002). Acute myocardial infarction in a young woman with severe ulcerative colitis. *International Journal of Cardiology*.

[98] Calderón R, Cruz-Correa MR, Torres EA (1998). Cerebral thrombosis associated with active Crohn’s disease. *Puerto Rico Health Sciences Journal*.

[99] Cognat E, Crassard I, Denier C, Vahedi K, Bousser MG (2011). Cerebral venous thrombosis in inflammatory bowel diseases: eight cases and literature review. *International Journal of Stroke*.

[100] Aloi M, Tromba L, Di Nardo G (2012). Premature subclinical atherosclerosis in pediatric inflammatory bowel disease. *The Journal of Pediatrics*.

[101] Yorulmaz E, Adali G, Yorulmaz H, Ulasoglu C, Tasan G, Tuncer I (2011). Metabolic syndrome frequency in inflammatory bowel diseases. *Saudi Journal of Gastroenterology*.

[102] Oussalah A, Guéant JL, Peyrin-Biroulet L (2011). Meta-analysis: hyperhomocysteinaemia in inflammatory bowel diseases. *Alimentary Pharmacology & Therapeutics*.

[103] Canero A, Parmeggiani D, Avenia N (2012). Thromboembolic tendency (TE) in IBD, (Inflammatory bowel disease) patients. *Annali Italiani di Chirurgia*.

[104] Roblin X, Germain E, Phelip JM (2006). Factors associated with hyperhomocysteinemia in inflammatory bowel disease: prospective study in 81 patients. *Revue de Medecine Interne*.

[105] McGowan CE, Jones P, Long MD, Barritt AS (2012). Changing shape of disease: nonalcoholic fatty liver disease in Crohn's disease-a case series and review of the literature. *Inflammatory Bowel Diseases*.

[106] Di Minno MN, Tufano A, Rusolillo A, Di Minno G, Tarantino G (2010). High prevalence of nonalcoholic fatty liver in patients with idiopathic venous thromboembolism. *World Journal of Gastroenterology*.

[107] Di Minno MN, Iervolino S, Peluso R (2012). Hepatic steatosis and disease activity in subjects with psoriatic arthritis receiving tumor necrosis factor-*α* blockers. *The Journal of Rheumatology*.

[108] Haapamäki J, Roine RP, Turunen U, Färkkilä MA, Arkkila PET (2011). Increased risk for coronary heart disease, asthma, and connective tissue diseases in inflammatory bowel disease. *Journal of Crohn’s and Colitis*.

[109] Sridhar ARM, Parasa S, Navaneethan U, Crowell MD, Olden K (2011). Comprehensive study of cardiovascular morbidity in hospitalized inflammatory bowel disease patients. *Journal of Crohn’s and Colitis*.

[110] Osterman MT, Yang YX, Brensinger C, Forde KA, Lichtenstein GR, Lewis JD (2011). No increased risk of myocardial infarction among patients with ulcerative colitis or Crohn’s disease. *Clinical Gastroenterology and Hepatology*.

[111] Dorn SD, Sandler RS (2007). Inflammatory bowel disease is not a risk factor for cardiovascular disease mortality: results from a systematic review and meta-analysis. *American Journal of Gastroenterology*.

[112] Di Minno MN, Iervolino S, Peluso R, Scarpa R, Di Minno G (2012). TNF-*α* blockers and carotid intima-media thickness: an emerging issue in the treatment of psoriatic arthritis. *Internal and Emergency Medicine*.

[113] Di Minno MN, Iervolino S, Peluso R, Scarpa R, Di Minno G (2011). Carotid intima-media thickness in psoriatic arthritis: differences between tumor necrosis factor-*α* blockers and traditional disease-modifying antirheumatic drugs. *Arteriosclerosis, Thrombosis, and Vascular Biology*.

[114] Bisoendial RJ, Stroes ESG, Tak PP (2009). Where the immune response meets the vessel wall. *Netherlands Journal of Medicine*.

[115] Di Minno MN, Iervolino S, Lupoli R (2012). Cardiovascular risk in rheumatic patients: the link between inflammation and atherothrombosis. *Seminars in Thrombosis and Hemostasis*.

[116] Gonzalez-Juanatey C, Llorca J, Amigo-Diaz E, Dierssen T, Martin J, Gonzalez-Gay MA (2007). High prevalence of subclinical atherosclerosis in psoriatic arthritis patients without clinically evident cardiovascular disease or classic atherosclerosis risk factors. *Arthritis Care and Research*.

[117] Sari I, Okan T, Akar S (2006). Impaired endothelial function in patients with ankylosing spondylitis. *Rheumatology*.

[118] Mathieu S, Joly H, Baron G (2008). Trend towards increased arterial stiffness or intima-media thickness in ankylosing spondylitis patients without clinically evident cardiovascular disease. *Rheumatology*.

[119] Caliskan M, Erdogan D, Gullu H (2008). Impaired coronary microvascular and left ventricular diastolic functions in patients with ankylosing spondylitis. *Atherosclerosis*.

[120] Malesci D, Niglio A, Mennillo GA, Buono R, Valentini G, La Montagna G (2007). High prevalence of metabolic syndrome in patients with ankylosing spondylitis. *Clinical Rheumatology*.

[121] Choe JY, Lee MY, Rheem I, Rhee MY, Park SH, Kim SK (2008). No differences of carotid intima-media thickness between young patients with ankylosing spondylitis and healthy controls. *Joint Bone Spine*.

[122] Di Minno MN, Tufano A, Ageno W, Prandoni P, Di Minno G (2012). Identifying high-risk individuals for cardiovascular disease: similarities between venous and arterial thrombosis in perspective. A 2011 update. *Internal and Emergency Medicine*.

[123] Ferrer I, Benavent G, Bastida G (2013). Peripheral arterial thromboembolism in Crohn's disease. *Gastroenterología y Hepatología*.

[124] Tabibian JH, Streiff MB (2012). Inflammatory bowel disease-associated thromboembolism: a systematic review of outcomes with anticoagulation versus catheter-directed thrombolysis. *Inflammatory Bowel Diseases*.

[125] Issa H, Al-Momen S, Bseiso B (2011). Thomboembolism in inflammatory bowel diseases: a report from Saudi Arabia. *Clinical and Experimental Gastroenterology*.

[126] Danese S, Papa A, Saibeni S, Repici A, Malesci A, Vecchi M (2007). Inflammation and coagulation in inflammatory bowel disease: the clot thickens—CME. *American Journal of Gastroenterology*.

[127] Kume K, Yamasaki M, Tashiro M, Yoshikawa I, Otsuki M (2007). Activations of coagulation and fibrinolysis secondary to bowel inflammation in patients with ulcerative colitis. *Internal Medicine*.

[128] Chiarantini E, Valanzano R, Liotta AA (1996). Hemostatic abnormalities in inflammatory bowel disease. *Thrombosis Research*.

[129] Weber P, Husemann S, Vielhaber H, Zimmer KP, Nowak-Göttl U (1999). Coagulation and fibrinolysis in children, adolescents, and young adults with inflammatory bowel disease. *Journal of Pediatric Gastroenterology and Nutrition*.

[130] van Bodegraven AA, Schoorl M, Linskens RK, Bartels PC, Tuynman HA (2002). Persistent activation of coagulation and fibrinolysis after treatment of active ulcerative colitis. *European Journal of Gastroenterology and Hepatology*.

[131] Ghosh S, Mackie MJ, McVerry BA, Galloway M, Ellis A, McKay J (1983). Chronic inflammatory bowel disease, deep-venous thrombosis and antithrombin activity. *Acta Haematologica*.

[132] Papa A, Scaldaferri F, Danese S (2008). Vascular involvement in inflammatory bowel disease: pathogenesis and clinical aspects. *Digestive Diseases*.

[133] Horowitz S, Binion DG, Nelson VM (2007). Increased arginase activity and endothelial dysfunction in human inflammatory bowel disease. *American Journal of Physiology*.

[134] Morowitz DA, Allen LW, Kirsner JB (1968). Thrombocytosis in chronic inflammatory bowel disease. *Annals of Internal Medicine*.

[135] Yüksel O, Helvaci K, Başar O (2009). An overlooked indicator of disease activity in ulcerative colitis: mean platelet volume. *Platelets*.

[136] Collins CE, Cahill MR, Newland AC, Rampton DS (1994). Platelets circulate in an activated state in inflammatory bowel disease. *Gastroenterology*.

[137] Irving PM, Macey MG, Shah U, Webb L, Langmead L, Rampton DS (2004). Formation of platelet-leukocyte aggregates in inflammatory bowel disease. *Inflammatory Bowel Diseases*.

[138] Bernstein CN, Wajda A, Blanchard JF (2005). The clustering of other chronic inflammatory diseases in inflammatory bowel disease: a population-based study. *Gastroenterology*.

[139] Kaufmann HJ, Taubin HL (1987). Nonsteroidal anti-inflammatory drugs activate quiescent inflammatory bowel disease. *Annals of Internal Medicine*.

[140] Bjarnason I, Hayllar J, MacPherson AJ, Russell AS (1993). Side effects of nonsteroidal anti-inflammatory drugs on the small and large intestine in humans. *Gastroenterology*.

[141] Kaufman HL, Fischer AH, Carroll M, Becker JM (1996). Colonic ulceration associated with nonsteroidal anti-inflammatory drugs: report of three cases. *Diseases of the Colon and Rectum*.

[142] Dissanayake AS, Truelove SC (1973). A controlled therapeutic trial of long term maintenance treatment of ulcerative colitis with sulphasalazine (Salazopyrin). *Gut*.

[143] Dick AP, Grayson MJ, Carpenter RG, Petrie A (1964). Controlled trial of sulphasalazine in the treatment of ulcerative colitis. *Gut*.

[144] Ferraz MB, Tugwell P, Goldsmith CH, Atra E (1990). Meta-analysis of sulfasalazine in ankylosing spondylitis. *Journal of Rheumatology*.

[145] Rodríguez-Bores L, Barahona-Garrido J, Yamamoto-Furusho JK (2007). Basic and clinical aspects of osteoporosis in inflammatory bowel disease. *World Journal of Gastroenterology*.

[146] Padovan M, Castellino G, Govoni M, Trotta F (2006). The treatment of the rheumatologicalmanifestations of the inflammatoryboweldiseases. *Rheumatology International*.

[147] de Keyser F, van Damme N, de Vos M, Mielants H, Veys EM (2000). Opportunities for immune modulation in the spondyloarthropathies with special reference to gut inflammation. *Inflammation Research*.

[148] Chen J, Liu C (2004). Methotrexate for ankylosing spondylitis. *Cochrane Database of Systematic Reviews*.

[149] Haibel H, Rudwaleit M, Braun J, Sieper J (2005). Six months open label trial of leflunomide in active ankylosing spondylitis. *Annals of the Rheumatic Diseases*.

[150] Peluso R, Atteno M, Iervolino S (2009). Methotrexate in the treatment of peripheral arthritis in ulcerative colitis. *Reumatismo*.

[151] van den Bosch F, Kruithof E, de Vos M, de Keyser F, Mielants H (2000). Crohn’s disease associated with spondyloarthropathy: effect of TNF-*α* blockade with infliximab on articular symptoms. *The Lancet*.

[152] Marzo-Ortega H, McGonagle D, O’Connor P, Emery P (2003). Efficacy of etanercept for treatment of Crohn’s related spondyloarthritis but not colitis. *Annals of the Rheumatic Diseases*.

[153] Iervolino S, Tramontano G, Lofrano M (2011). Etanercept nel trattamento dell’artrite enteropatica associata a malattia di Crohn: descrizione di un caso clinico. *Reumatismo*.

[154] Neuman MG, Nanau RM (2012). Inflammatory bowel disease: role of diet, microbiota, life style. *Translational Research*.

[155] Veerappan GR, Betteridge J, Young PE (2012). Probiotics for the treatment of inflammatory bowel disease. *Current Gastroenterology Reports*.

[156] Karimi O, Peña AS (2005). Probiotics in arthralgia and spondyloarthropathies in patients with inflammatory bowel disease. Prospective randomized trials are necessary. *Revista Espanola de Enfermedades Digestivas*.

[157] Rudwaleit M, Baeten D (2006). Ankylosing spondylitis and bowel disease. *Best Practice and Research: Clinical Rheumatology*.

[158] Behm BW, Bickston SJ (2008). Tumor necrosis factor-alpha antibody for maintenance of remission in Crohn’s disease. *Cochrane Database of Systematic Reviews*.

[159] Gandhi S, Narula N, Marshall JK, Farkouh M (2012). Are patients with inflammatory bowel disease at increased risk of coronary artery disease?. *The American Journal of Medicine*.

